# BIODIESEL: Cultivating Alternative Fuels

**DOI:** 10.1289/ehp.115-a86

**Published:** 2007-02

**Authors:** Charles W. Schmidt

Back in the early 1990s, U.S. farmers took note of the first Gulf War, rising energy prices, and a huge glut of excess soybean oil sitting in tanks around the country, and they saw an opportunity. Soybean oil, they reasoned, could be refined to make biodiesel, an alternative fuel source. In Europe—where diesel fuel powers up to half the entire vehicle fleet—biodiesel was being produced in industrial quantities using rapeseed oil. Why not do the same with soybean oil, the farmers asked, and turn existing surpluses into an energy commodity?

The idea caught on; in 1992, the National SoyDiesel Development Board was formed to study biodiesel production based on the European model. In 1994, when the organization’s name was changed to the National Biodiesel Board (NBB), fuels produced from soybean oil amounted to barely a few thousand gallons a year. But ten years later, that volume had grown to 25 million gallons, mainly due to the efforts of the NBB. The addition of a tax subsidy worth up to $1 per gallon, which took effect in January 2005, sent demand for the fuel soaring. Joe Jobe, the NBB’s chief executive officer, says at least 200 million gallons were sold in 2006. Assuming existing and emerging facilities operate at full capacity, U.S. biodiesel production capacity could reach 1.5 billion gallons in 2007, he predicts.

Biodiesel, useable in any diesel engine, is now a key player in the alternative fuels market. Produced by industrial facilities that turn out millions of gallons annually, and also by smaller manufacturers that make it from used cooking grease, biodiesel could do much to reduce our reliance on foreign oil, experts say. “Long-term, we estimate it could produce a volume equal to about twenty-five percent of today’s on-highway diesel fuel use,” says Robert McCormick, a principal engineer at the DOE National Renewable Energy Laboratory (NREL).

McCormick stresses that biodiesel can’t replace petroleum entirely. Although diesel powers most of the commercial trucks, ships, and farm equipment in the United States, roughly 95% of the passenger fleet here runs on gasoline. Even if the passenger fleet were to shift entirely to diesel, U.S. agriculture couldn’t produce enough feed-stock to meet its needs, he says. According to NREL’s calculations, published in the June 2004 report *Biomass Oil Analysis: Research Needs and Recommendations*, agricultural capacity in the United States would probably limit production to at most 10 billion gallons of pure biodiesel a year, unless manufacturers used new higher-yield feedstocks, such as algae.

Still, according to Jonathan Cogan, a spokesman for the federal Energy Information Administration, the United States consumed more than 40 billion gallons of diesel fuel in 2005 alone. The possibility that biodiesel could substitute for up to a quarter of that amount is significant, McCormick emphasizes. “Biodiesel will be part of a multi-pronged approach to replacing imported petroleum,” he says.

## A Brief History

The diesel engine was invented in 1892 by engineer Rudolf Diesel. Diesel engines differ significantly from standard gas engines. Where gas engines ignite vaporized fuel in a cylinder using a spark plug, diesel engines compress air in a cylinder, making it so hot that when fuel hits the air, it explodes. That process converts fuel to energy more efficiently than spark plug designs, giving diesel engines greater fuel economy.

Early diesel engines ran exclusively on vegetable oil. But in the 1920s, the feed-stock shifted to petroleum distillates refined from crude oil during gasoline production. But while so-called petrodiesel was cheaper and more plentiful than vegetable oil, it was also lighter and less viscous. Automakers had to modify engine designs accordingly, and vegetable oil as a fuel source was sidelined for decades.

Then in 1973, the Arab oil embargo sent crude oil prices through the roof. With gas and diesel suddenly four times more expensive than before, interest in biofuels returned. But there was a dilemma: pure vegetable oil was too thick for modern diesel engines; it plugged injection systems and didn’t spray evenly into compression cylinders. Short of going back to older engine designs, two options remained: either heat the oil with an onboard system to make it less viscous (the method used by today’s “Greasecars,” which run on straight fryer grease), or make the oil’s molecules smaller.

The latter option led to biodiesel. Most producers chose a manufacturing method called transesterification, which the South Africans used to make fuel from vegetable oil before World War II. With that process, refiners mix the oil with alcohol in the presence of a catalyst, usually sodium hydroxide. The alcohol and fatty acids react, creating biodiesel and a by-product of glycerin. The alcohol used is usually methanol, yielding a biodiesel consisting of fatty acid methyl esters.

Today, most biodiesel produced worldwide is made by transesterification. Soybean oil accounts for nearly 90% of the biodiesel produced in the United States, although any kind of vegetable oil or animal fat is suitable. Most scientists dismiss earlier suggestions that biodiesel requires more fossil fuel energy to make (in terms of chemical inputs, labor, transportation, and other factors) than it generates as fuel. What many consider to be the definitive analysis, described in a 1998 DOE/USDA report titled *An Overview of Biodiesel and Petroleum Diesel Life Cycles*, concluded that biodiesel generates 3.2 times more energy than is required to produce it.

Bill Holmberg, who chairs the Biomass Coordinating Council at the American Council on Renewable Energy, suggests that over time the balance will shift even more in biodiesel’s favor. “If we put our minds to it, we can reduce the amount of fossil energy going into biodiesel production, perhaps not to zero, but substantially nonetheless,” he says. “It’s a matter of conservation and applying existing technology wisely.”

## Biodiesel in Action

Pure biodiesel, called B100, can generally be used only at higher temperatures: as it reaches the freezing point of water, B100 gels up and causes engine trouble. To use it in cold weather, drivers must install special heating systems to keep the fuel warm. Even pure diesel can gel up in extreme cold, Jobe says, and biodiesel blends at any level can exacerbate that problem. As an added hindrance, B100 has strong solvent properties that liberate rust and other engine contaminants, which plug filters and fuel injectors. (With repeated use, however, B100 and biodiesel blends “clean” engines of these contaminants, which become less troublesome with time.)

To avoid these problems, most drivers use blends of B100 and petrodiesel mixed at varying ratios. A blend called B20—20% pure biodiesel—has long been the biggest seller, but according to Jobe, lower blends have begun to overtake B20. Those containing 2% and 5% biodiesel—designated B2 and B5, respectively—now drive much of the growth in the market, he says. That’s because small amounts of biodiesel acts as a lubricant in the ultra-low sulfur diesel (ULSD) fuels now emerging to meet heightened pollution standards in some states, protecting the engine against wear.

Supporters insist biodiesel’s benefits outweigh its inconveniences, for not just energy security but also the environment. Numerous studies show that compared to petrodiesel, B20 emits at least 10% less particulate matter, carbon monoxide, and total hydrocarbons. The relevant data are summarized in a 2006 NREL report titled *Effects of Biodiesel Blends on Vehicle Emissions*. Unlike fossil fuels—which contain carbon from underground sources—biodiesel contains carbon from plants that were recently alive and drawing carbon from the atmosphere. For that reason, burning it doesn’t add more carbon dioxide to the atmosphere than what was already there.

What’s more, biodiesel contains 11% oxygen by weight, which enhances fuel combustion, and reduces the amount of carcinogenic soot that diesel engines spew into the air. Diesel engines have traditionally had a bad reputation when it comes to pollution. Petrodiesel can contain a lot of sulfur, which generates sulfate-based particulates that cause acid rain and contributes to health problems ranging from respiratory illness to cancer. For that reason, some states—including Maine, California, Massachusetts, New York, and Vermont—have banned sales of diesel-powered passenger vehicles altogether. (Vehicles purchased elsewhere can still be registered in those states, however.) Since 15 October 2006, most diesel sold in the United States is ULSD, which contains a maximum of 15 ppm sulfur, and all model year 2007 diesel vehicles for highway use must use this fuel. Biodiesel does one better, however, because it contains no sulfur.

## Locking Horns over NO_x_

However, McCormick points out that biodiesel emits questionable amounts of nitrogen oxides (NO_x_)—air pollutants that mix with sunlight to form smog, a respiratory irritant. “Many studies show small increases in NO_x_ from B20, but many other studies show decreases,” he says. “It’s hard to know what’s correct from the data we have today.”

In the 2002 draft technical report *A Comprehensive Analysis of Biodiesel Impacts on Exhaust Emissions*, the EPA reviewed a range of engine testing studies and concluded that, on average, NO_x_ levels emitted by soy-derived B20 are 2% higher than those produced by petrodiesel. That’s worrisome because diesel engines already emit high levels of NO_x_, and smog is a major health and environmental problem. But these results were challenged by NREL scientists, who claim the EPA relied too heavily on data for just one engine design—the test-bed engine —thus biasing their results. NREL’s own review, described in *Effects of Biodiesel Blends on Vehicle Emissions*, suggests that NO_x_ emissions from biodiesel can vary depending on feedstock, engine type, and testing methods.

Scott Gordon, a chemist and founder of Green Technologies, a small biodiesel producer in Winooski, Vermont, emphasizes that most U.S. studies have employed test-bed engines, which don’t mimic NO_x_ emissions under real-world conditions. Moreover, catalytic converters that normally remove NO_x_ from gas engines can be used on compression engines that burn ULSD fuel, he says. “Sulfur poisons catalytic converters, and that’s why diesel engines traditionally haven’t used them,” he explains. “But with [ultra-] low sulfur diesel fuel, the engine industry is shifting towards catalytic converters, and that could lower NO_x_ emissions dramatically.”

When asked about the issue, an EPA spokesperson replied, “Biodiesel fuels can achieve significant [particulate matter] reductions. EPA is currently working with stakeholders to understand all the potential impacts that NO_x_ emissions from biodiesel may have.”

Still, the EPA’s findings prompted the Texas Commission on Environmental Quality (TCEQ) to propose a ban on biodiesel in 110 counties. According to its website, the TCEQ assumes, based on EPA figures, that NO_x_ emissions from B100 are 10% higher than allowed under the state’s new diesel standards. Biodiesel blends follow accordingly—emissions from B20, for example, are assumed by the TCEQ to be 2% higher than state standards allow. The ban wouldn’t necessarily be iron-clad; producers would be able to conduct independent testing and sell biodiesel if their NO_x_ emissions are low enough. But that testing costs over $100,000, which is more than many producers can afford.

The proposed ban was scheduled to go into effect on 31 December 2006. Three weeks before that deadline, however, the TCEQ granted biodiesel a one-year reprieve. This extension will allow ongoing studies to reach final conclusions and give the industry a chance to continue testing formulations to comply with the Texas low-emission diesel standards.

According to Gordon, the Texas ban, if implemented, could negatively influence biodiesel’s growth in other states with borderline NO_x_ compliance, which include Vermont, his company’s home base. “It definitely sets a precedent,” he says.

## Incentives for Growth

Ironically, Texas’s proposed ban came as many states are pushing for greater biodiesel use. In September 2005 Minnesota implemented a new rule that diesel sold in-state must contain at least 2% B100. A similar law, passed in Washington State in March 2006, mandates a 2% B100 minimum for diesel sales now, increasing to 5% as state biodiesel production rises.

The federal government’s tax credit is also a proven incentive for biodiesel growth. A product of NBB and other stakeholder lobbying, the credit applies mainly to fuel distributors and blenders. For every percentage of B100 blended in fuel, a penny gets deducted from the federal excise tax for diesel, which is 24.4¢ per gallon. A gallon of B100—100% biodiesel—therefore qualifies for a $1 tax credit. Likewise, a gallon of B20 qualifies for a 20¢ deduction, which reduces the distributor’s tax obligation to 4.4¢.

However, the full credit applies only to B100 made with pure, “first-use” vegetable oil. A gallon made with used cooking oil—a commodity known in the industry as yellow grease—qualifies for only a 50¢ credit. So while the credit has been a boon for big producers and the soy industry, it’s been less so for local producers like Gordon who rely on donated yellow grease as feedstock to supply a mainly off-road market geared toward farm equipment and home heating oil.

Biodiesel’s rapid growth hasn’t come without consequences. In 2006, a nationwide NREL survey of 38 blending facilities—meaning facilities that mix biodiesel for distribution—found unacceptably high levels of total glycerin in up to one-third of samples tested, indicating the feedstock fat had not been completely converted. That meant the samples were therefore out of compliance with quality standards issued by ASTM International, the body that governs standards for industrial materials.

McCormick attributes the quality lapses to “sheer incompetence,” and says bound glycerin (the glycerin in unconverted or partly converted fat) can plug fuel filters in cold weather, making engines difficult to start. “It creates an immediate problem for the user,” he says. “And these guilty blenders may be claiming the tax credit, for which they are eligible only if they pass ASTM standards.” According to McCormick, NREL plans to issue a report on the subject later this year.

Jobe stresses that the NBB is concerned about quality, and suggests that lapses come from an explosive rise in demand. “With this kind of growth you’re going to find large numbers of new producers coming online who haven’t gotten all their quality control procedures in place,” he says. “We’re taking aggressive measures to promote quality, because even one bad player can give the whole industry a bad name.”

## Food for Fuel

Biodiesel’s rapid expansion—combined with that of other biofuels such as ethanol—has led some to worry that fuel generation could divert agriculture from food, leaving some people hungry. Margarine manufacturers in Germany and France, where nearly 860 million gallons of biodiesel were produced in 2005, already complain they’re being sidelined by crop diversions to the fuel industry. Could that signal a more dramatic competition to come as biodiesel production accelerates?

Most experts say no. Grant Kimberley, who directs market development for the Iowa Soybean Association, says that in the United States, the soybean oil used for biodiesel so far comes from existing surpluses, meaning the industry hasn’t diverted any oil into the fuel market yet. And Jobe emphasizes that by making soybean oil more valuable, biodiesel production lessens the pressure on solid soy meal (the portion with the protein) to generate dollars for the industry. “And that will allow farmers to get more money for the whole bean while driving down the cost of meal that generates protein for livestock feed,” he says. “The only people who might go hungry are those who subsist on french fries, margarine, and Italian dressing.”

Moreover, experts anticipate that future biodiesel feedstocks will generate higher oil yields than soybeans. Whereas soybeans yield 18–20% oil, other crops produce much more; the oil yield from canola, for instance, tops 40%. Jake Stewart, vice president for strategic development at Organic Fuels, a Houston, Texas–based refinery that made 30 million gallons of biodiesel in 2006 (making it the largest producer in Texas and the third largest producer in the United States), says the industry has barely scratched the potential when it comes to higher-yield crops.

While declining to speak specifically about his company’s leanings in this area, Stewart suggests the industry will look to completely different species with higher oil yields, such as shrub trees. The biggest contender of all, Stewart says, is algae, which has an oil yield of up to 50%. “That’s the only feedstock with the potential to really displace petroleum in this country,” he says. Whereas soybeans generate roughly 50 gallons of biodiesel per acre, algal species can produce up to 8,000 gallons per acre per year, according to Michael Briggs, a PhD candidate in physics who investigates biodiesel production at the University of New Hampshire. This makes them the most promising potential feedstock by far.

The trick is to somehow grow algae in systems that allow producers to control production. Open ponds are problematic, says Briggs, because it’s hard to control species distribution. To make a uniform product, manufacturers need a system that grows just one selected species, without infiltration by others.

Briggs says the favored approach employs closed bioreactors that keep unwanted species out while allowing for precise control of light, water quality, and nutrient inputs. In one blue-sky scenario, producers could install bioreactors throughout the country and grow algae with nutrients obtained from wastewater treatment facilities, he says. A total of 15,000 square miles, equal to about 12.5% of the area occupied by the Sonora Desert, could generate 140 billion gallons of biodiesel—enough to replace nearly all the petroleum used for transportation in the United States today (assuming gas-driven cars switched to diesel technology).

For another comparison, Briggs notes that 15,000 square miles works out to about 9.5 million acres—far less than the 442 million acres devoted to cropland or the 586 million acres devoted to grassland pasture for livestock grazing in the United States, according to figures from the USDA.

But beyond the United States, land diversions for biodiesel are more problematic. Indonesian rainforests are being burned now to free up acreage for palm trees, a biodiesel feedstock that yields more than 600 gallons of B100 per acre. Rampant clearing in the tropics could have disastrous consequences: rainforests absorb carbon dioxide and help mitigate the effects of global warming. Moreover, according to a 5 December 2006 article in *The Wall Street Journal*, forest fires set to clear land for palm trees on Borneo have covered the capital city of Pontianak with smoke and added to the smog that already blankets much of Southeast Asia.

One anonymous source, who plans to construct a major biodiesel production facility in Texas using imported palm oil for 50% of the feedstock, says tropical land clearing for fuel is a “prolific” practice. He adds, “We don’t want our crude palm oil coming from areas that used to be rainforest; our biodiesel comes from sustainable production. But this is like the diamond trade—there’s a right way and a wrong way to do things.”

Meanwhile, the lure of growing markets for biodiesel fueled by subsidized demand could prove irresistible to developing world distributors willing to slash rainforests for palm oil, even as they claim sustainability in public. Without more oversight, farming for biodiesel could exacerbate deforestation worldwide, and obviate the fuel’s climate benefits, while contributing to erosion, air pollution, loss of biodiversity, and other environmental threats.

Ultimately, biodiesel could offer a ray of hope for a world squeezed by declining oil supplies, pollution, and global warming. But it’s also an industry beset with growing pains and the threat of unsustainable production, particularly in the developing world. If one thing is certain, it’s that biodiesel is a technology to watch. Before long, it could be the fuel of choice for millions.

## Figures and Tables

**Figure:**
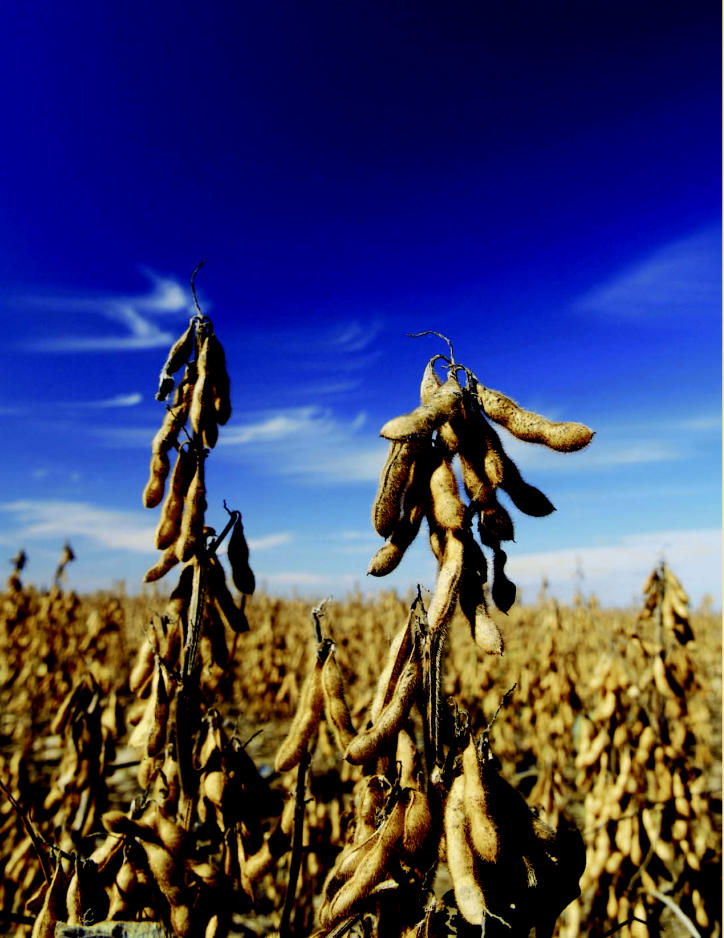


**Figure:**
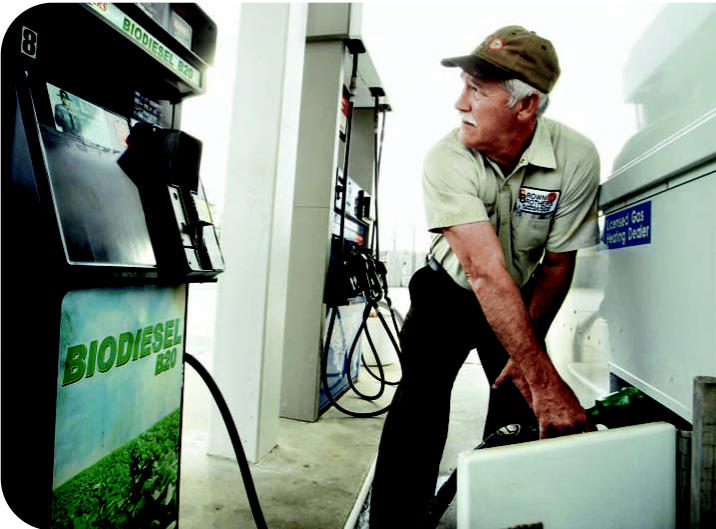
A new way to go An Exxon station in Durham, North Carolina, offers a biodiesel mixture made from petroleum and organic feed sources such as soybeans, cooking oil, and animal fats.

**Figure:**
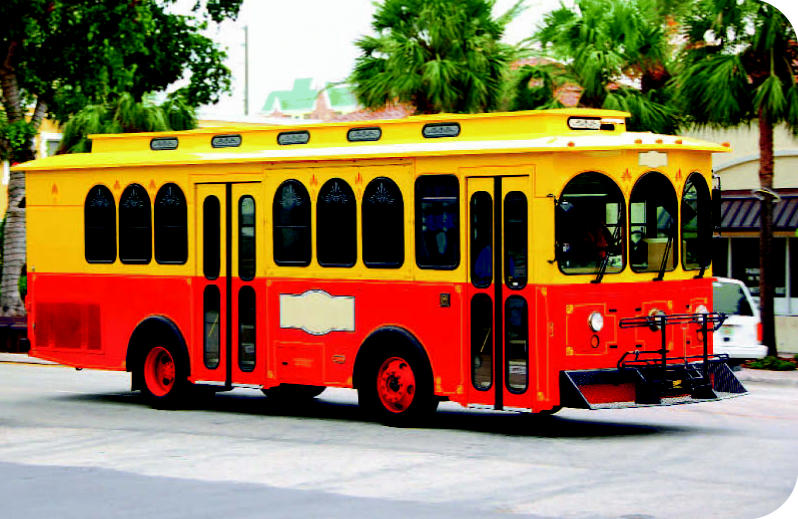
A brighter future? The Sun Trolley public transportation fleet in Fort Lauderdale, Florida, is one of the first in the United States to begin using biodiesel for its entire fleet.

**Figure:**
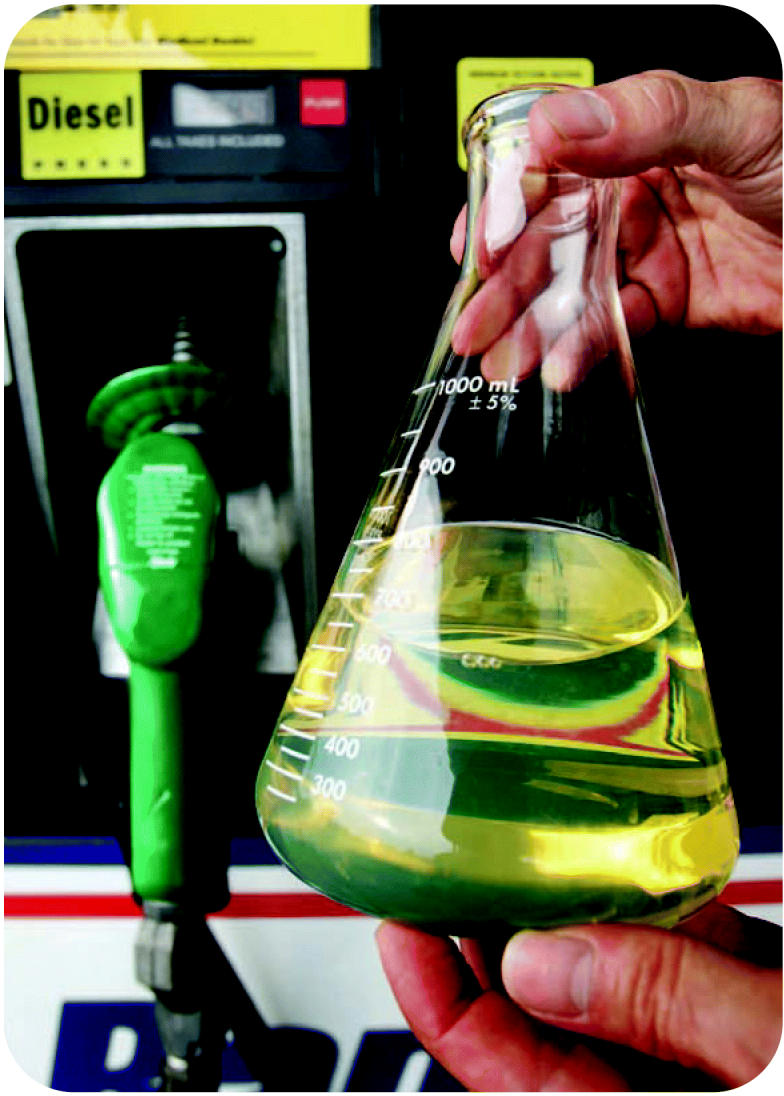
A healthier mix A sample of B20 fuel containing 20% biodiesel and 80% standard diesel. Biodiesel made from chemically altered vegetable oil burns more cleanly than traditional diesel fuel.

**Figure:**
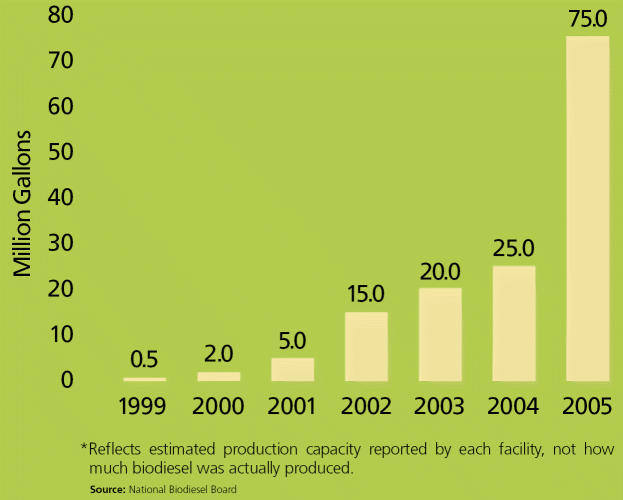
U.S. Biodiesel Production Capacity*

